# Expression of Adipose MicroRNAs Is Sensitive to Dietary Conjugated Linoleic Acid Treatment in Mice

**DOI:** 10.1371/journal.pone.0013005

**Published:** 2010-09-27

**Authors:** Pilar Parra, Francisca Serra, Andreu Palou

**Affiliations:** Department of Fundamental Biology and Health Sciences, University of the Balearic Islands and CIBER de Fisiopatología de la Obesidad y Nutrición (CIBEROBN), Palma de Mallorca, Baleares, Spain; Sapienza University of Rome, Italy

## Abstract

**Background:**

Investigation of microRNAs (miRNAs) in obesity, their genetic targets and influence by dietary modulators is of great interest because it may potentially identify novel pathways involved in this complex metabolic disorder and influence future therapeutic approaches. This study aimed to determine whether miRNAs expression may be influenced by conjugated linoleic acid (CLA), currently used to induce fat loss.

**Methodology/Principal Findings:**

We determined retroperitoneal adipose tissue (rWAT) expression of five miRNAs related to adipocyte differentiation (miRNA-143) and lipid metabolism (miRNA-103 and -107) and altered in obesity (miRNA-221 and -222), using the TaqMan®MicroRNA Assay (Applied-Biosystems). In the first experiment, mice were fed with a standard fat diet and orally treated with sunflower oil (control group) and 3 or 10 mg CLA/day for 37 days. In the second experiment, mice were fed with a high fat diet for 65 days. For the first 30 days, mice received the same doses of CLA described above and, from that time onwards, animals received a double dose. Results showed that expression of selected miRNAs was modified in response to CLA treatment and metabolic status. Interestingly, a strong correlation was observed between miR-103 and -107 expression, as well as miR-221 and -222 in both experiments. Moreover, changes in miRNAs expression correlated with several adipocyte gene expressions: miR-103 and -107 correlated with genes involved in fatty acid metabolism whereas miR-221 and miR-222 correlated with the expression of adipocytokines. Regarding the minor changes observed in miR-143 expression, no differences in expression of adipogenic markers were observed.

**Conclusions/Significance:**

Although elucidating the functional implications of miRNAs is beyond the scope of this study, these findings provide the first evidence that miRNAs expression may be influenced by dietary manipulation, reflecting or even contributing to the new metabolic state originated by CLA treatment.

## Introduction

In the last decade, a novel class of RNA regulatory genes known as miRNAs has been found to introduce a new level of gene regulation in eukaryotes [1]. miRNAs are transcribed as long primary-miRNAs (pri-miRNA) that encode a single miRNA or a cluster of miRNA species. Genomic mapping has revealed that pri-miRNA species are encoded within noncoding genomic sequences as well as in introns or, less frequently exons, of protein-coding genes. The processing of pri-miRNAs is initiated in the nucleus and is further continued in the cytoplasm giving rise to a 19–22 bp long mature miRNA. The mature miRNA is then incorporated into a protein complex, the RNA-induced silencing complex, where the miRNA strand anneals to the 3′ untranslated regions of target mRNAs to promote mRNA degradation or translational repression, but in some cases, increases its translational activity [2]. The versatility of miRNA-mediated gene regulation is evidenced by the finding that individual miRNAs can target hundreds of genes while individual mRNAs can be targeted by multiple miRNAs, allowing for enormous complexity and flexibility in their regulatory potential [3]–[6].

Many miRNAs are conserved across species and intervene in a variety of physiological processes including growth, differentiation, development and energy metabolism. The first evidence for participation of miRNAs in lipid metabolism came from a study in *Drosophila melanogaster*, in which miR-14 was identified as necessary for normal fat metabolism [7]. In mammals, miRNAs have been shown to modulate adipocyte differentiation [8]–[11], cholesterol and lipid homeostasis in liver [12], [13] as well as insulin secretion and signalling [14], [15]. Recent papers have observed an association between the expression of specific miRNAs and obesity [16]–[19] supporting the fact that miRNAs may play a role in the pathological development of obesity and also leading to the hypothesis that miRNAs may represent a new class of adipogenic regulators with potential therapeutic interest against obesity. However, no effects on the impact of supplementation with nutrients that modulate body composition on the expression of miRNAs have been described yet.

Among nutrients, CLA, which refers to a group of positional and geometric isomers of linoleic acid, has been reported to reduce fat deposition both in animals (reviewed in [20]) and, to a lesser extent, in humans (reviewed in [21]), and in consequence, is used as dietary supplement for weight loss.

We have recently shown that moderate doses of an equimolar mix of the two main active isomers are associated to lower fat accretion in mice both under standard-fat [22] and high-fat diet [23] without inducing liver steatosis and keeping insulin sensitivity. In a further step, the purpose of the present study was to assess whether miRNAs could play a role in the novel steady state induced by CLA. Consequently, expression levels of selected miRNAs (miR-143, miR-103, miR-107, miR-221 and miR-222) which seem to be related to adipose biology were studied in adipose tissue of mice treated with CLA. We found that their expression was modified in response to CLA treatment and metabolic status, furthermore strong correlations were observed between their expressions and/or with several adipocyte gene expressions.

## Results

### Adipose tissue miRNAs expression

**Figure 1:**
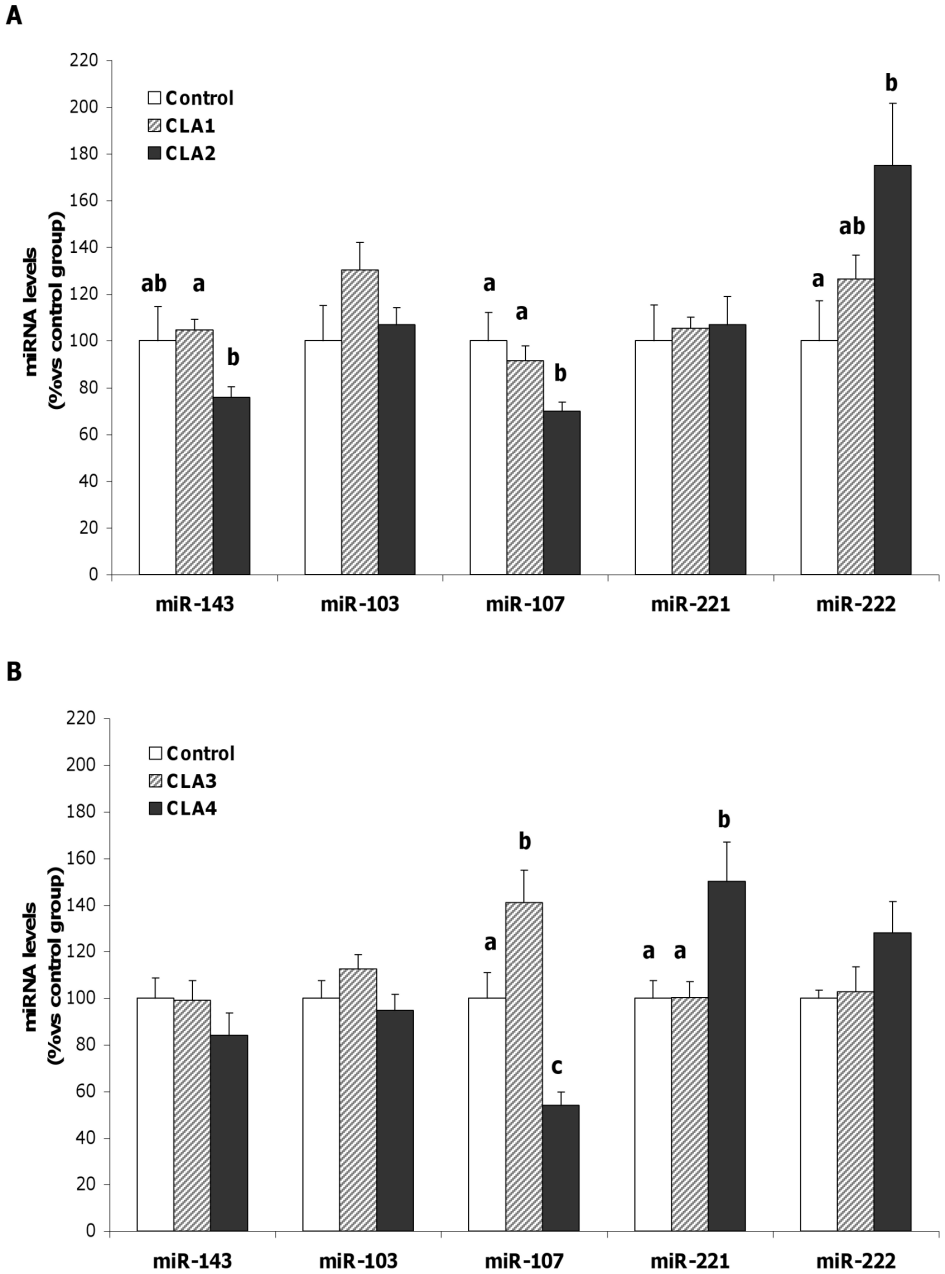
Relative expression of miRNAs of mice maintained on different dietary regimes and treated with CLA. After CLA treatment, expression levels of miRNAs were measured by real-time PCR in rWAT of mice fed a standard-fat diet (**A**) or a high-fat diet (**B**). Data are means ± SEM of n = 10–11 mice in Exp1 and n = 6–8 mice in Exp2. Mean values with unlike letters are significantly different (P<0.05); one-factor ANOVA followed by LSD test.

CLA was shown to modulate the expression of selected miRNA in adipose tissue in a specific manner, reflecting the impact of the type of diet, the metabolic status and the CLA dose (Figure 1). The lowest dose of CLA did not cause any change in miR-143 expression, either in experiment 1 (Exp1) or experiment 2 (Exp2). However, the highest doses of CLA produced a decrease in each experimental design (by 20%) although only reached statistically significance in Exp1, when comparing CLA treated groups (P<0.05). No changes in miR-103 were observed by CLA treatment in either of the experiments. In Exp1, miR-107 showed a dose-dependent decrease in its expression, which attained statistical significance at the highest dose (CLA2) with respect to both CLA1 and control group (P<0.05). Interestingly, under high-fat diet (Exp2) miR-107 showed a different expression pattern depending on the dose. A 40% increase (P<0.05) with respect to the control value was found with the lowest dose (CLA3 group) in contrast with the 46% decrease (P<0.01) with respect to the control group that was observed at the highest dose (CLA4). miR-221 expression was not affected in Exp1 and only the highest dose of CLA in Exp2 (CLA4) produced a significant increase of miR-221 with respect to both CLA3 and control groups (P<0.01). Concerning miR-222 expression, a tendency to increase expression with the dose was seen in Exp1, although only the 75% increase produced in CLA2 group was statistically significant compared to the control group (P<0.01). In Exp2, miR-222 expression increased with the highest dose (CLA4 group) but did not reach statistical significance.

### Relationship within miRNAs expression

We further tested the hypothesis that miRNAs are expressed in a coordinated manner as a response to the remodelation of adipose tissue by CLA treatment. Data concerning the correlations within their expression levels confirmed that the expression of miRNAs was tightly correlated, particularly under normal-fat feeding (Exp1) (Table 1). In fact, expression of all miRNAs tested was significant and highly correlated within themselves, except for the case of miR-222 which was only correlated with miR-103 and miR-221. This strong association was partially lost in Exp2, with CLA treatment under high-fat feeding, in which the only correlations maintained were between miR-103 and miR-143; mir103 and miR-107; and miR-221 and miR-222 (Table 1).

**Table 1:** Correlations within miRNAs expression in mice treated with CLA under standard diet or a high-fat diet.

	*Exp1 (standard-fat diet)*					*Exp2 (high-fat diet)*				
	miR-143	miR-103	miR-107	miR-221	miR-222	miR-143	miR-103	miR-107	miR-221	miR-222
**miR-143**		0.677**	0.915**	0.622**	0.249		0.754**	0.418	-0.033	0.123
**miR-103**			0.696**	0.654**	0.393*			0.563**	0.183	0.312
**miR-107**				0.589**	0.197				-0.269	0.011
**miR-221**					0.787**					0.831**

The comparison between miRNAs expression levels in rWAT was done by the Pearson Chi-square test.

Statistical significance (2-tailed)

*P<0.05,

**P<0.01.

### Expression of adipogenic marker genes

Since CLA treatments seem to alter common adipocyte gene expression, we used RT-PCR to analyze expression levels of some adipogenic marker genes such as glucose transporter type 4 (Glut4), lipoprotein lipase (Lpl), peroxisome proliferator activator receptor gamma 2 (PPARγ2) and CCAAT/enhancer binding protein alpha (C/EBPα). In Exp1 under a stardard-fat diet (Table 2) no differences in Lpl or the transcription factor PPARγ2 were observed by CLA treatment. Glut4 showed a statistically significant increase with the lowest dose (CLA1) with respect to both CLA2 and control group (P<0.01). C/EBPα slightly decreased with the highest dose but only reached statistical significance when comparing with the lowest dose (CLA1 group) (P<0.01). CLA treatment did not produce changes in the expression of these genes in Exp2 (Table 2).

**Table 2:** Adipogenic gene expression of mice treated with CLA under standard diet or a high-fat diet.

	*Exp1 (standard-fat diet)*			*Exp2 (high-fat diet)*		
	Control	CLA1	CLA2	Control	CLA3	CLA4
**Glut4**	100±5^a^	129±7^b^	84±10^a^	100±12	117±10	112±10
**Lpl**	100±9	105±5	102±9	100±5	90±6	100±9
**C/EBPα**	100±6^ab^	111±9^a^	81±6^b^	100±8	120±11	100±9
**PPAR**γ**2**	100±7	107±6	87±8	100±6	96±5	85±8

The expression levels of adipocyte differentiation markers (Glut4, Lpl, C/EBPα, and PPARγ2) were analyzed by RT-PCR.

Data are means ± SEM of 8–12 mice in Exp1 and of 7 mice in Exp2. Means in a row without a common letter differ, P<0.05; one-factor ANOVA followed by LSD test.

### Correlations between miRNAs and adipocyte gene expression

We further analyzed the expression of more adipocyte related genes with the aim to determine the potential relationship between the expression of the selected miRNAs and the adipocyte phenotype. In Exp1, miR-143 correlated positively with adiponectin (r = 0.357, P<0.05) and leptin expression (r = 0.358, P<0.05); miR-103 was correlated with two key markers of lipid metabolism, fatty acid synthase (Fasn, r = 0.378, P<0.05) and muscle carnitine palmitoyltransferase 1b (Cpt1b, r = 0.404, P<0.05); miR-107 correlated with genes involved in fatty acid oxidation such as uncoupling protein 2 (Ucp2, r = −0.339, P<0.05) and Cpt1b (r = 0.467, P<0.01) as well as with C/EBPα (r = 0.370, P<0.05) (Table 3). miR-222 showed significant correlations with an array of genes related to adipocyte metabolism including Glut4 (r = −0.400, P<0.05); genes related to lypolisis such as hormone sensitive lipase (HSL, r = −0.379, P<0.05) and patatin-like phospolipase domain containing 2 (Pnpla2, r = −0.346, P<0.05); lipogenesis such as PPARγ2 (r = −0.396, P<0.05), Fasn (r = −0.416, P<0.05), stearoyl-Coenzyme A desaturase 1 (Scd1, r = −0.392, P<0.05); fatty acid oxidation such as Ucp2 (r = 0.525, P<0.01); and adipocytokines: adiponectin (r = −0.385, P<0.05) and tumor necrosis factor alpha (TNFα, r = 0.644, P<0.01) (Table 3). miR-221 was also specifically correlated with the expression of TNFα (r = 0.385, P<0.05) (Table 3). In Exp2, miR-107 expression in adipocytes was significantly correlated with the expression of Lpl (r = −0.428, P<0.05), PPARα (r = 0.494, P<0.05) and TNFα (r = −0.510, P<0.05), additionally under high-fat feeding, the correlation found by CLA under normal-fat feeding between miR-107 and Cpt1b (r = 0.466, P<0.05) remained significant (Table 4). The high number of correlations with miR-222 was not found in Exp2. Correlation found for miR-221 and TNFα in Exp1 was maintained in Exp2 (r = 0.434, P<0.05) and a significant negative correlation between adiponectin and miR-221 (r = −0.444, P<0.05) was also identified (Table 4). Significant and non-significant correlations of both experiments were showed in the Table S1 (Exp1) and Table S2 (Exp2).

**Table 3:** Significant correlations between adipocyte genes expression and miRNAs in mice fed with a standard-fat diet and treated with CLA.

	*Experiment 1 (standard-fat diet)*				
	miR-143	miR-103	miR-107	miR-221	miR-222
**Glut4**					−0.400*
**C/EBPα**			0.370*		
**PPARγ2**					−0.396*
**Fasn**		0.378*			−0.416*
**Scd1**					−0.392*
**Pnpla2**					−0.346*
**HSL**					−0.379*
**Cpt1b**		0.404*	0.467**		
**Ucp2**			−0.339*		0.525**
**Adiponectin**	0.357*				−0.385*
**Leptin**	0.358*				
**TNFα**				0.385*	0.644**

The comparison between miRNAs and adipocyte gene expression in rWAT was done by the Pearson Chi-square test. Statistical significance (2-tailed)

*P<0.05,

**P<0.01.

**Table 4:** Significant correlations between adipocyte genes expression and miRNAs in mice fed with a high-fat diet and treated with CLA.

	*Experiment 2 (high-fat diet)*				
	miR-143	miR-103	miR-107	miR-221	miR-222
**Lpl**			−0.428*		
**PPARα**			0.494*		
**Cpt1b**			0.466*		
**Adiponectin**				−0.444*	
**TNFα**			−0.510*	0.434*	

The comparison between miRNAs and adipocyte gene expression in rWAT was done by the Pearson Chi-square test. Statistical significance (2-tailed)

*P<0.05,

**P<0.01.

## Discussion

We have previously reported that the use of moderate doses of CLA reduces body fat accretion in mice maintained on different dietary regimes (Exp1 and Exp2) [22], [23]. In both experiments, the rWAT was the most sensitive to the effects of CLA, decreasing up to 67% under CLA treatment in comparison with controls. Therefore, this adipose tissue was selected to assess whether CLA treatment could produce any impact on miRNAs expression levels. Several mechanisms by which CLA decreases fat mass have been proposed such as increased energy expenditure, regulation of genes encoding for enzymes involved in lipid synthesis, promotion of adipocyte apoptosis or decreased preadipocyte proliferation and differentiation (reviewed in [20], [24]). The complex response to CLA in adipose tissue is likely to take place through a CLA-mediated modulation of major metabolic regulators which are not totally known. In agreement with this assumption, the present study has demonstrated that changes in miRNA expression occur in adipose tissue after CLA treatment, suggesting a novel level of regulation by which CLA might exert its effects.

Emerging evidence suggests that specific miRNAs contribute to the regulation of adipocyte differentiation and in consequence may play a key role in the pathological development of obesity [8], [11], [25]. As potential targets of the action of CLA, we focused the study on five selected miRNAs (miR-143, miR-103, miR-107, miR-221 and miR-222) which, to a certain extent, have been shown to be involved in adipocyte differentiation and/or associated with obesity.

MiR-143 was the first miRNA associated with regulation of adipocyte differentiation. Its expression increases in differentiating adipocytes, and antisense oligonucleotides against miR-143 inhibit human cultured adipocyte differentiation and lead to a decrease in triglyceride accumulation and the downregulation of PPARγ2, adipocyte fatty acid binding protein and Glut4 [8], although this has not been found in 3T3-L1 cells [9]. Later on it was demonstrated that in mesenteric fat, miR-143 expression is upregulated in mice fed a high-fat diet and this was associated with elevated body and mesenteric fat weight as well as with markers of adipocyte differentiation [16]. Accordingly, a decrease in expression of miR-143 by CLA would justify a lower adipogenic capacity and, would therefore contribute to the decrease of fat stores observed in adipose tissue with CLA treatment. However, our data showed small changes, if any, in the expression levels of miR-143. Only the highest CLA dose assayed in each experiment caused a slight decrease in miR-143 expression, but not significantly different from controls. In accordance, gene expression of adipogenic markers such as Glut4, Lpl, PPARγ2 and C/EBPα was not decreased by CLA treatment in either of the experiments, suggesting that the body fat lowering effect of CLA observed in our conditions was unlikely to be a consequence of a reduction in the adipogenic capacity.

Another aspect of interest concerns miR-103 and miR-107 regulation by CLA. Some miRNAs originate from introns of known genes; in this case, they can be co-transcribed with the ‘parent’ mRNAs or independently [26]–[29]. For instance, miR-107, miR-103-1 and miR-103-2 reside in the sense orientation in intron 5 of the three panthothenate kinase (PANK) gene family members, PANK1, PANK3 and PANK2, respectively. A computational study predicted that this family of miRNAs affects multiple target genes in metabolic pathways in a manner that points to a coordinated function with the PANK genes, which are central players in regulating intracellular Co-enzyme A [30]–[32]. This has been further supported by experimental data showing that induction of miR-103 and -107 during *in vitro* adipogenesis is accompanied by induction of the respective PANK gene expression [11]. We found high and significant correlations between the expression of miR-103 and -107 in both experimental settings suggesting coordinated expression of both miRNAs. Interestingly, in Exp1, miR-107 was correlated with markers of the oxidative pathway while miR-103 was correlated with the expression of two limiting enzymes in fatty acid metabolism, such as Fasn and Cpt1b, suggesting that CLA intake might contribute, to some extent, to modulate the role of these two miRNAs in channeling Acil-CoA derivatives to lipogenesis and/or to oxidation. However, in Exp2 this situation was different. Although expression levels of miR-103 did not change by CLA treatment, the associations of its expression with phenotype markers of adipocytes were not found. Concerning expression of miR-107, CLA altered the profile of its expression in both experiments and this resulted in novel associations with adipocyte gene expression, only the association with Cpt1b was sustained in both experimental designs. The above data point out that the potential role of modulation of miRNAs *in vivo* is more complex than *in vitro* assays and, in addition, is affected by a number of external factors such as obesity, dietary treatment, metabolic status, etc.

Additionally, a recent report described an inverse relation of several miRNAs expression during adipogenesis and obesity. A set of miRNAs (including miR-103, miR-107 and miR-143) are induced during adipogenesis, which may play a role in accelerating fat cell development, and then be downregulated in the obese state [11]. Conversely, another set of miRNAs follows the opposite response pattern, for example miR-222 and miR-221, which are decreased during adipogenesis but upregulated in obese adipocytes [11]. In agreement with this pattern, we found a high and significant correlation between the expressions of these two miRNAs in both experiments, but contrary to the above, the miRNAs did not follow a parallel regulation of their expression under CLA treatment.

Although little is known about the adipocyte biology of both miRNAs, treatment of differentiated 3T3-L1 adipocytes for 24 h with TNFα has been shown to induce expression of both miRNAs, which has been associated with the role of TNFα, as a major macrophage-produced cytokine involved in chronic inflammation [11], being largely responsible for inducing insulin resistance in obese adipose tissue [33]. Our data point out that CLA was able to counteract the joint expression profile of these two miRNAs observed in the obese state, where both miRNAs increase simultaneously [11] and this is therefore associated with adipocyte dysfunction and insulin resistance [11], which was not observed in our study using moderate doses of CLA [22], [23]. In fact, one of the phenotypic characteristics of the treatment with CLA in the present study concerns the maintenance of glucose-insulin homeostasis, which is unaltered in Exp1 [22] and, despite moderate hyperglucemia and hyperinsulinemia in Exp2, insulin sensitivity, assayed by the revised quantitative insulin sensitivity check index is preserved in this experimental setting [23].

**Figure 2:**
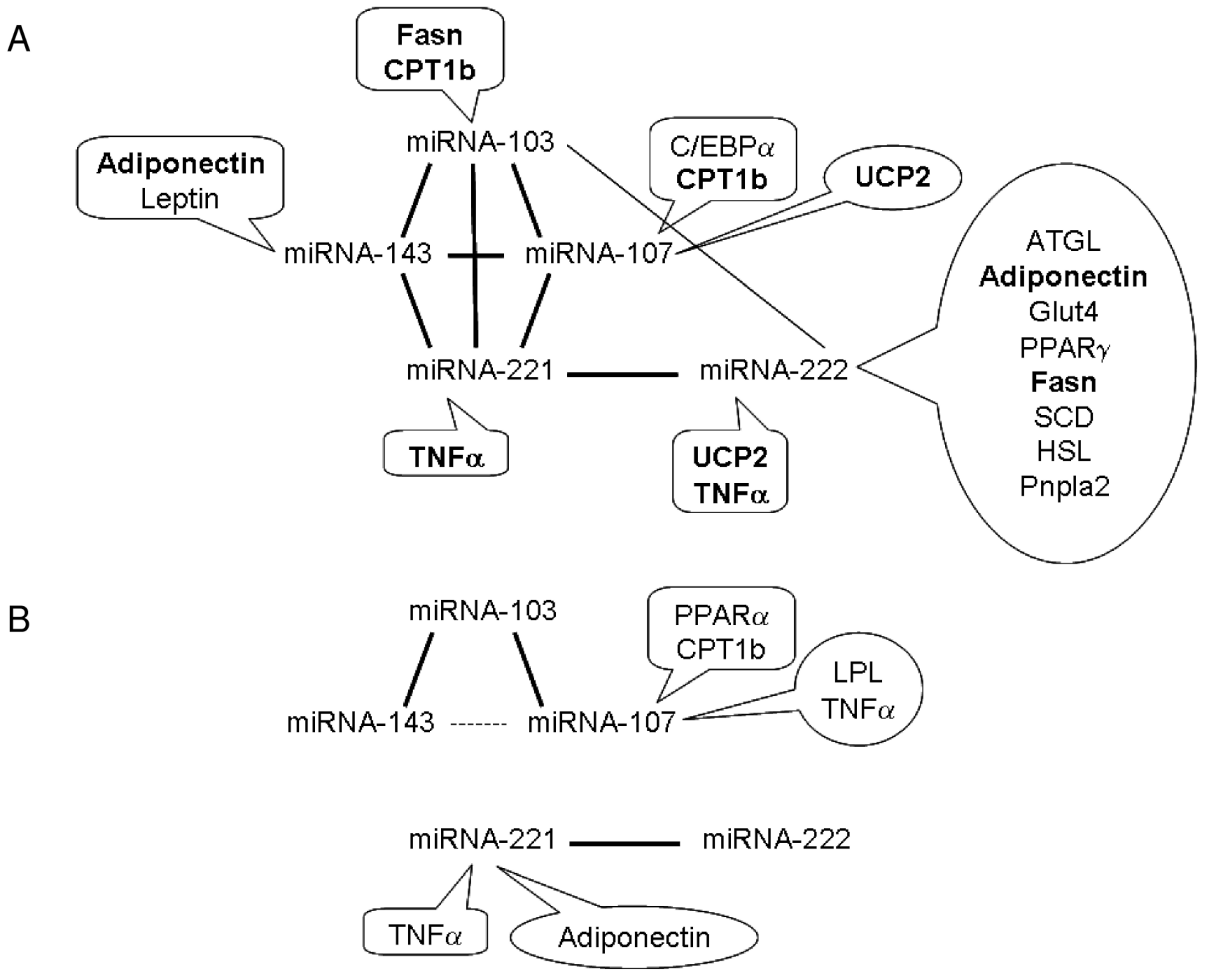
Schematic representation of the correlations between the expression of adipose miRNAs and of key markers of adipocyte metabolism. Correlations were found in mice fed a standard-fat diet (A) and a high-fat diet (B). Two levels of statistical significance have been considered and the wider lines indicate the strongest correlation. The discontinuous line shows a non statistically significant correlation (p = 0.418). Positive correlations are listed in squared forms and the negative ones in round forms. The bold names indicate adipocyte markers that are correlated with more than one miRNA.

A schematic representation of the correlations found (Figure 2), helps to show the interrelationships which take place in adipose tissue between the expression of microRNAs and mRNAs under CLA treatment. Interestingly, the main outcome is that the five miRNAs constitute a highly correlated core of signals, particularly under standard fat diet (Exp1). In addition, CLA treatment during high fat feeding (Exp2) shows a dissociation between the pair of miRNAs 221/222 and the others. Furthermore, the expression of a number of markers of adipocyte function (including markers of fatty acid synthesis and transport) correlates with the expression of the miRNAs. All in all, the most relevant seems to be the role of the adipokines adiponectin and TNFα, whose expression keeps the correlation linked to the pair of miRNAs 221/222, irrespective of the different set up between Exp1 and Exp2, therefore reflecting the impact of CLA on adipose miRNAs.

In this context, we have previously found decreased production of leptin (accompanying fat loss) which goes hand in hand with decreased expression of adiponectin [22], [23]. We have suggested that the decrease in body fat accretion induced by CLA treatment under these specific conditions, decreases leptin and goes hand in hand with lower adiponectin levels, reaching a novel set point between these two circulating adipocytokines, which is associated with the maintenance of insulin sensitivity and a general trend of decreased expression of inflammatory markers in adipose tissue [22] such as monocyte chemotactic protein-1 (MCP1), interleukin-6 (IL-6), inducible nitric oxide synthase (iNOS) and TNFα [22], [23].

Although elucidating the functional implications of miRNAs is beyond the scope of this study, our data show that specific miRNAs are sensitive to dietary manipulation and reflect the metabolic changes that take place under CLA treatment. These results are the basis for novel ways in the field of research into CLA. Research into miRNAs in obesity, their genetic targets and influence by dietary modulators is of great interest because it may potentially identify new pathways involved in this complex metabolic disorder and influence future approaches to the treatment of obesity [19], [34]. However, further studies will be needed to understand the role of miRNAs in CLA action and to assess the potential of miRNA profiles to predict nutritional status in which CLA might trigger beneficial effects.

## Materials and Methods

### Animals and diets

Male C57BL/6J mice from Charles River (Barcelona, Spain) weighing 20±0.2 g were housed in groups of four in plastic cages and maintained on a 12-h light:dark cycle at 22°C. These mice were used in two independent experiments with different dietary regimes. In Exp1 animals were fed *ad libitum* with a standard diet (D12450B, Research Diets Inc, New Brunswick) which contains 10% calorie content as fat (25 g/100 g soybean oil and 20 g/100 g lard), 70% calorie content as carbohydrate (315 g/100 g corn starch, 35 g/100 g maltrodextrin 10, 350 g/100 g sucrose and 50 g/100 g cellulose) and the remaining 20% as protein (casein and L-cystine). In Exp2 animals were fed *ad libitum* with a high fat diet (D12451, Research Diets Inc, New Brunswick) which contains 45% calorie content as fat (25 g/100 g soybean oil and 177.5 g/100 g lard), 35% calorie content as carbohydrate (72.8 g/100 g corn starch, 100 g/100 g maltrodextrin 10, 172.8 g/100 g sucrose and 50 g/100 g cellulose)and the remaining 20% as protein (casein and L-cystine). Food intake and body weight were recorded every three days during the experiments. Fresh food was provided to the mice biweekly.

All experimental procedures were performed according to both national and institutional guidelines for animal care and use.

### CLA Treatment

Mice from both experiments were orally treated with CLA. The CLA used was Tonalin ® TG 80 derived from safflower oil (kindly provided by Cognis). Tonalin is composed of triglycerides containing approximately 80% conjugated linoleic acids with a 50∶50 ratio of the active CLA isomers *cis-*9, *trans-*11 and *trans-*10, *cis-*12.

In Exp1, mice were randomly assigned to three experimental groups (n = 12 each group): sunflower oil (control), CLA1 or CLA2 group. Two different doses of CLA were assayed in this study: CLA1 (0.15 g CLA/kg body weight) and CLA2 (0.50 g CLA/kg body weight), taking as a reference the weight of the animals at the beginning of the experiment. Therefore, animals received a daily amount of Tonalin equivalent to 3 mg CLA/animal in CLA1 group and 10 mg/animal in CLA2 group for 37 days. Control animals received an isocaloric dose of commercially available sunflower olive oil.

In Exp2, mice were also assigned to three experimental oral treatments (n = 8 each group): sunflower oil (control), CLA3 or CLA4 group for 65 days. For the first 30 days the same doses of CLA used in Exp1 were assayed, then after 30 days of treatment and until the end of the experiment, the dose of each group was doubled. Therefore, animals received a daily amount of Tonalin equivalent to 6 mg CLA/animal in CLA3 and 20 mg/animal in CLA4 groups for the last 35 days of treatment. An adequate amount of commercial sunflower oil was given to the animals to achieve an isocaloric load between groups.

### Sacrifice and tissue sampling

Mice from Exp1 were sacrificed under feeding conditions whereas mice from Exp2 were fasted for 10 h before sacrifice. Mice were anaesthetized by intraperitoneal injection of a mixture of xilacine (10 mg/kg body weight) and ketamine (100 mg/kg body weight). rWAT was rapidly removed, weighed, rinsed with saline containing 0.1% diethyl pyrocarbonate (Sigma, Madrid, Spain), frozen with nitrogen liquid, and stored at −70°C.

### RNA extraction and gene expression analyses

Total RNA from rWAT was extracted using Tripure Reagent (Roche Diagnostic Gmbh, Mannheim, Germany) according to the manufacturer's instructions. Isolated RNA was quantified using the NanoDrop®Spectrophotometer ND-1000 and its integrity confirmed by agarose gel electophoresis.

Gene expression was assessed by Real Time-PCR performed in an Applied Biosystems StepOnePlus™ Real-Time PCR System (Applied Biosystems). For miRNAs measurements, RNA was first reverse-transcribed and then amplified using the specific primers and probes provided with the TaqMan® MicroRNA Reverse Transcription kit (Applied Biosystems). To determine the adipocyte genes of interest, total RNA was reverse transcribed to cDNA as previously described [22] and amplified with gene-specific primers and Power SYBR Green PCR Master Mix (Applied Biosystems). Primer sequences are listed in Table S3. Relative quantification of a target gene was calculated based on efficiency and the crossing point deviation of an unknown sample versus a control, and expressed in comparison to a reference gene used to normalize cDNA (U6 small nuclear RNA and 18S ribosomal RNA for miRNAs and mRNAs target genes, respectively). Details of the protocol are available in Text S1.

### Statistical analysis

Data are presented as means ± SEM. One-factor ANOVA was used to determine the significance of the differences between groups. If there was a significant difference, a Least Significant Difference (LSD) test was used to determine the particular effect that caused that difference. P<0.05 was statistically significant, and different superscripts discriminate differences between groups. Linear relationships between key variables were tested using Pearson's correlation coefficients. The analysis was performed using the SPSS program for Windows version 16 (SPSS, Chicago, IL, USA).

## Supporting Information

Table S1 The comparison between miRNAs and adipocyte gene expression in rWAT was done by the Pearson Chi-square test. Statistical significance (2-tailed) P<0.05 (*), P<0.01 (**).
(0.06 MB DOC)

Table S2 The comparison between miRNAs and adipocyte gene expression in rWAT was done by the Pearson Chi-square test. Statistical significance (2-tailed) P<0.05 (*), P<0.01 (**).
(0.06 MB DOC)

Table S3 Target genes: C/EBPalpha, CCAAT/enhancer binding protein alpha; Cpt1b, muscle carnitine palmitoyltransferase 1b; Fasn, fatty acid synthase; Glut4, glucose transporter type 4; HSL, hormone sensitive lipase; Lpl, lipoprotein lipase; Pnpla2, patatin-like phospolipase domain containing 2; PPAR, peroxisome proliferator activator receptor; Scd1, stearoyl-Coenzyme A desaturase 1; Ucp, uncoupling protein. 18S was used for normalization.
(0.03 MB DOC)

Text S1 (0.03 MB DOC)

## References

[1] Lewis BP, Burge CB, Bartel DP (2005). Conserved seed pairing, often flanked by adenosines, indicates that thousands of human genes are microRNA targets.. Cell.

[2] Vasudevan S, Tong Y, Steitz JA (2007). Switching from repression to activation: microRNAs can up-regulate translation.. Science.

[3] Baek D, Villen J, Shin C, Camargo FD, Gygi SP (2008). The impact of microRNAs on protein output.. Nature.

[4] Selbach M, Schwanhausser B, Thierfelder N, Fang Z, Khanin R (2008). Widespread changes in protein synthesis induced by microRNAs.. Nature.

[5] Shalgi R, Lieber D, Oren M, Pilpel Y (2007). Global and local architecture of the mammalian microRNA-transcription factor regulatory network.. PLoS Comput Biol.

[6] Krek A, Grun D, Poy MN, Wolf R, Rosenberg L (2005). Combinatorial microRNA target predictions.. Nat Genet.

[7] Xu P, Vernooy SY, Guo M, Hay BA (2003). The Drosophila microRNA Mir-14 suppresses cell death and is required for normal fat metabolism.. Curr Biol.

[8] Esau C, Kang X, Peralta E, Hanson E, Marcusson EG (2004). MicroRNA-143 regulates adipocyte differentiation.. J Biol Chem.

[9] Kajimoto K, Naraba H, Iwai N (2006). MicroRNA and 3T3-L1 pre-adipocyte differentiation.. Rna.

[10] Sun T, Fu M, Bookout AL, Kliewer SA, Mangelsdorf DJ (2009). MicroRNA let-7 regulates 3T3-L1 adipogenesis.. Mol Endocrinol.

[11] Xie H, Lim B, Lodish HF (2009). MicroRNAs induced during adipogenesis that accelerate fat cell development are downregulated in obesity.. Diabetes.

[12] Krutzfeldt J, Stoffel M (2006). MicroRNAs: a new class of regulatory genes affecting metabolism.. Cell Metab.

[13] Esau C, Davis S, Murray SF, Yu XX, Pandey SK (2006). miR-122 regulation of lipid metabolism revealed by in vivo antisense targeting.. Cell Metab.

[14] He A, Zhu L, Gupta N, Chang Y, Fang F (2007). Overexpression of micro ribonucleic acid 29, highly up-regulated in diabetic rats, leads to insulin resistance in 3T3-L1 adipocytes.. Mol Endocrinol.

[15] Poy MN, Eliasson L, Krutzfeldt J, Kuwajima S, Ma X (2004). A pancreatic islet-specific microRNA regulates insulin secretion.. Nature.

[16] Takanabe R, Ono K, Abe Y, Takaya T, Horie T (2008). Up-regulated expression of microRNA-143 in association with obesity in adipose tissue of mice fed high-fat diet.. Biochem Biophys Res Commun.

[17] Nakanishi N, Nakagawa Y, Tokushige N, Aoki N, Matsuzaka T (2009). The up-regulation of microRNA-335 is associated with lipid metabolism in liver and white adipose tissue of genetically obese mice.. Biochem Biophys Res Commun.

[18] Kloting N, Berthold S, Kovacs P, Schon MR, Fasshauer M (2009). MicroRNA expression in human omental and subcutaneous adipose tissue.. PLoS One.

[19] Ortega FJ, Moreno-Navarrete JM, Pardo G, Sabater M, Hummel M (2010). MiRNA expression profile of human subcutaneous adipose and during adipocyte differentiation.. PLoS One.

[20] Wang YW, Jones PJ (2004). Conjugated linoleic acid and obesity control: efficacy and mechanisms.. Int J Obes Relat Metab Disord.

[21] Whigham LD, Watras AC, Schoeller DA (2007). Efficacy of conjugated linoleic acid for reducing fat mass: a meta-analysis in humans.. Am J Clin Nutr.

[22] Parra P, Serra F, Palou A (2010). Moderate doses of conjugated linoleic acid isomers mix contribute to lowering body fat content maintaining insulin sensitivity and a noninflammatory pattern in adipose tissue in mice.. J Nutr Biochem.

[23] Parra P, Palou A, Serra F (2010). Moderate doses of conjugated linoleic acid reduce fat gain, maintain insulin sensitivity without impairing inflammatory adipose tissue status in mice fed a high-fat diet.. Nutr Metab (Lond).

[24] House RL, Cassady JP, Eisen EJ, McIntosh MK, Odle J (2005). Conjugated linoleic acid evokes de-lipidation through the regulation of genes controlling lipid metabolism in adipose and liver tissue.. Obes Rev.

[25] Lin Q, Gao Z, Alarcon RM, Ye J, Yun Z (2009). A role of miR-27 in the regulation of adipogenesis.. Febs J.

[26] Baskerville S, Bartel DP (2005). Microarray profiling of microRNAs reveals frequent coexpression with neighboring miRNAs and host genes.. Rna.

[27] Rodriguez A, Griffiths-Jones S, Ashurst JL, Bradley A (2004). Identification of mammalian microRNA host genes and transcription units.. Genome Res.

[28] Ying SY, Lin SL (2006). Current perspectives in intronic micro RNAs (miRNAs).. J Biomed Sci.

[29] Lin SL, Miller JD, Ying SY (2006). Intronic MicroRNA (miRNA).. J Biomed Biotechnol.

[30] Wilfred BR, Wang WX, Nelson PT (2007). Energizing miRNA research: a review of the role of miRNAs in lipid metabolism, with a prediction that miR-103/107 regulates human metabolic pathways.. Mol Genet Metab.

[31] Rock CO, Calder RB, Karim MA, Jackowski S (2000). Pantothenate kinase regulation of the intracellular concentration of coenzyme A.. J Biol Chem.

[32] Vallari DS, Jackowski S, Rock CO (1987). Regulation of pantothenate kinase by coenzyme A and its thioesters.. J Biol Chem.

[33] Cawthorn WP, Sethi JK (2008). TNF-alpha and adipocyte biology.. FEBS Lett.

[34] Heneghan HM, Miller N, Kerin MJ (2009). Role of microRNAs in obesity and the metabolic syndrome.. Obes Rev.

