# Supplementary material for: Expression of Adipose MicroRNAs Is Sensitive to Dietary Conjugated Linoleic Acid Treatment in Mice
Source: PLoS One. 2010 Sep 27;5(9):e13005. doi: 10.1371/journal.pone.0013005 (PMC2946340; doi:10.1371/journal.pone.0013005)
Supplement: Table S1 — The comparison between miRNAs and adipocyte gene expression in rWAT was done by the Pearson Chi-square test. Statistical significance (2-tailed) P<0.05 (*), P<0.01 (**). (0.06 MB DOC) [file pone.0013005.s001.doc]

**Table S1.** Pearson correlation coefficients between expression levels of adipocyte markers and miRNAs in mice fed with a standard-fat diet and treated with CLA

| *Experiment 1 (standard-fat diet)* | | | | | | |
| --- | --- | --- | --- | --- | --- | --- |
|  |  | **miR-143** | **miR-103** | **miR-107** | **miR-221** | **miR-222** |
| **Lpl** | Pearson's correlation | ,082 | ,028 | ,034 | -,090 | -,154 |
|  | Sig. (bilateral) | ,642 | ,874 | ,848 | ,613 | ,384 |
| **Pnpla2** | Pearson's correlation | -,027 | ,150 | ,014 | -,319 | -,346* |
|  | Sig. (bilateral) | ,879 | ,391 | ,934 | ,066 | ,045 |
| **C/EBPα** | Pearson's correlation | ,311 | ,341 | ,370* | ,002 | -,329 |
|  | Sig. (bilateral) | ,078 | ,052 | ,034 | ,990 | ,066 |
| **PPARγ2** | Pearson's correlation | ,136 | ,082 | ,169 | -,311 | -,396* |
|  | Sig. (bilateral) | ,436 | ,638 | ,331 | ,073 | ,020 |
| **SREBP1c** | Pearson's correlation | ,075 | ,112 | ,171 | -,240 | -,231 |
|  | Sig. (bilateral) | ,668 | ,520 | ,326 | ,171 | ,189 |
| **Scd1** | Pearson's correlation | ,035 | ,242 | ,003 | -,256 | -,392* |
|  | Sig. (bilateral) | ,846 | ,168 | ,986 | ,151 | ,024 |
| **Fasn** | Pearson's correlation | ,122 | ,378* | ,173 | -,216 | -,416* |
|  | Sig. (bilateral) | ,499 | ,030 | ,337 | ,236 | ,018 |
| **Ucp2** | Pearson's correlation | -,284 | ,030 | -,339* | ,179 | ,525** |
|  | Sig. (bilateral) | ,098 | ,865 | ,046 | ,311 | ,001 |
| **PPARα** | Pearson's correlation | ,009 | ,184 | ,009 | -,087 | -,067 |
|  | Sig. (bilateral) | ,958 | ,305 | ,960 | ,634 | ,716 |
| **Cpt1b** | Pearson's correlation | ,292 | ,404* | ,467** | -,023 | -,281 |
|  | Sig. (bilateral) | ,099 | ,020 | ,006 | ,902 | ,119 |
| **Cpt1a** | Pearson's correlation | -,104 | ,083 | -,089 | ,103 | ,035 |
|  | Sig. (bilateral) | ,559 | ,640 | ,616 | ,567 | ,849 |
| **Adiponectin** | Pearson's correlation | ,357* | ,171 | ,275 | -,062 | -,385* |
|  | Sig. (bilateral) | ,035 | ,326 | ,110 | ,727 | ,025 |
| **Leptin** | Pearson's correlation | ,358* | -,098 | ,274 | -,075 | -,229 |
|  | Sig. (bilateral) | ,041 | ,586 | ,122 | ,684 | ,208 |
| **TNFα** | Pearson's correlation | -,192 | ,164 | -,176 | ,385* | ,644** |
|  | Sig. (bilateral) | ,268 | ,346 | ,313 | ,025 | ,000 |
| **HSL** | Pearson's correlation | -,014 | ,219 | ,094 | -,204 | -,379* |
|  | Sig. (bilateral) | ,941 | ,246 | ,623 | ,290 | ,043 |
| **Glut4** | Pearson's correlation | ,275 | ,225 | ,290 | -,153 | -,400* |
|  | Sig. (bilateral) | ,135 | ,224 | ,113 | ,420 | ,028 |
