# Supplementary material for: Expression of Adipose MicroRNAs Is Sensitive to Dietary Conjugated Linoleic Acid Treatment in Mice
Source: PLoS One. 2010 Sep 27;5(9):e13005. doi: 10.1371/journal.pone.0013005 (PMC2946340; doi:10.1371/journal.pone.0013005)
Supplement: Table S2 — The comparison between miRNAs and adipocyte gene expression in rWAT was done by the Pearson Chi-square test. Statistical significance (2-tailed) P<0.05 (*), P<0.01 (**). (0.06 MB DOC) [file pone.0013005.s002.doc]

**Table S2.** Pearson correlation coefficients between expression levels of adipocyte markers and miRNAs in mice fed with a high-fat diet and treated with CLA

| *Experiment 2 (high-fat diet)* | | | | | | |
| --- | --- | --- | --- | --- | --- | --- |
|  |  | **miR-143** | **miR-103** | **miR-107** | **miR-221** | **miR-222** |
| **Lpl** | Pearson's correlation | ,110 | ,040 | -,428* | ,053 | ,002 |
|  | Sig. (bilateral) | ,625 | ,860 | ,047 | ,816 | ,993 |
| **Pnpla2** | Pearson's correlation | ,236 | ,102 | -,108 | ,029 | -,051 |
|  | Sig. (bilateral) | ,302 | ,661 | ,642 | ,902 | ,831 |
| **C/EBPα** | Pearson's correlation | ,075 | ,139 | -,043 | -,258 | -,165 |
|  | Sig. (bilateral) | ,740 | ,536 | ,848 | ,246 | ,475 |
| **PPARγ2** | Pearson's correlation | ,414 | ,322 | ,168 | -,297 | -,297 |
|  | Sig. (bilateral) | ,062 | ,155 | ,467 | ,190 | ,203 |
| **SREBP1c** | Pearson's correlation | -,175 | -,411 | -,103 | -,243 | -,291 |
|  | Sig. (bilateral) | ,437 | ,057 | ,648 | ,277 | ,201 |
| **Scd1** | Pearson's correlation | -,023 | ,089 | ,002 | -,157 | -,281 |
|  | Sig. (bilateral) | ,918 | ,693 | ,994 | ,485 | ,218 |
| **Fasn** | Pearson's correlation | ,122 | ,234 | ,338 | -,136 | -,028 |
|  | Sig. (bilateral) | ,589 | ,294 | ,124 | ,547 | ,902 |
| **Ucp2** | Pearson's correlation | -,107 | -,028 | -,401 | ,355 | ,150 |
|  | Sig. (bilateral) | ,636 | ,903 | ,064 | ,105 | ,517 |
| **PPARα** | Pearson's correlation | ,099 | -,010 | ,494* | -,318 | -,005 |
|  | Sig. (bilateral) | ,662 | ,966 | ,019 | ,149 | ,983 |
| **Cpt1b** | Pearson's correlation | ,025 | -,017 | ,466* | -,410 | -,160 |
|  | Sig. (bilateral) | ,916 | ,942 | ,038 | ,072 | ,512 |
| **Cpt1a** | Pearson's correlation | -,142 | -,214 | ,246 | ,223 | ,291 |
|  | Sig. (bilateral) | ,530 | ,340 | ,271 | ,318 | ,200 |
| **Adiponectin** | Pearson's correlation | ,169 | ,037 | ,029 | -,444* | -,409 |
|  | Sig. (bilateral) | ,453 | ,869 | ,899 | ,038 | ,066 |
| **Leptin** | Pearson's correlation | ,131 | ,107 | ,027 | -,269 | -,326 |
|  | Sig. (bilateral) | ,562 | ,635 | ,904 | ,225 | ,149 |
| **TNFα** | Pearson's correlation | -,205 | -,250 | -,510* | ,434* | ,287 |
|  | Sig. (bilateral) | ,359 | ,262 | ,015 | ,043 | ,208 |
| **HSL** | Pearson's correlation | ,340 | ,146 | ,353 | -,358 | -,279 |
|  | Sig. (bilateral) | ,121 | ,518 | ,107 | ,102 | ,220 |
| **Glut4** | Pearson's correlation | ,239 | -,051 | -,024 | ,030 | ,177 |
|  | Sig. (bilateral) | ,284 | ,821 | ,915 | ,895 | ,442 |
