# Supplementary material for: Expression of Adipose MicroRNAs Is Sensitive to Dietary Conjugated Linoleic Acid Treatment in Mice
Source: PLoS One. 2010 Sep 27;5(9):e13005. doi: 10.1371/journal.pone.0013005 (PMC2946340; doi:10.1371/journal.pone.0013005)
Supplement: Table S3 — Target genes: C/EBPalpha, CCAAT/enhancer binding protein alpha; Cpt1b, muscle carnitine palmitoyltransferase 1b; Fasn, fatty acid synthase; Glut4, glucose transporter type 4; HSL, hormone sensitive lipase; Lpl, lipoprotein lipase; Pnpla2, patatin-like phospolipase domain containing 2; PPAR, peroxisome proliferator activator receptor; Scd1, stearoyl-Coenzyme A desaturase 1; Ucp, uncoupling protein. 18S was used for normalization. (0.03 MB DOC) [file pone.0013005.s003.doc]

**Table S3.** Gene-specific primer sequences used in real-time PCR amplification.

| **Gene** | **Forward sequence (5’ → 3’)** | **Reverse sequence (5’ → 3’)** |
| --- | --- | --- |
| *C/EBPα* | AGGTGCTGGAGTTGACCAGT | CAGCCTAGAGATCCAGCGAC |
| *Cpt1b* | GCAAACTGGACCGAGAAGAG | CCTTGAAGAAGCGACCTTTG |
| *Fasn* | TTCGGTGTATCCTGCTGTCC | TGGGCTTGTCCTGCTCTAAC |
| *Glut4* | GGCATGCGTTTCCAGTATGT | GCCCCTCAGTCATTCTCATC |
| *HSL* | TCACGCTACATAAAGGCTGCT | CCACCCGTAAAGAGGGAACT |
| *Lpl* | CCTGATGACGCTGATTTTGT | TATGCTTTGCTGGGGTTTTC |
| *Pnpla2* | TGTGGCCTCATTCCTCCTAC | AGCCCTGTTTGCACATCTCT |
| *PPARα* | CGTTTGTGGCTGGTCAAGTT | AGAGAGGACAGATGGGGCTC |
| *PPARγ* | AGACCACTCGCATTCCTTTG | TCGCACTTTGGTATTCTTGG |
| *Scd1* | GGAAATGAACGAGAGAAGGTG | CCGAAGAGGCAGGTGTAGAG |
| *Ucp2* | GGTCGGAGATACCAGAGCAC | ATGAGGTTGGCTTTCAGGAG |
| *TNFα* | CGTCGTAGCAAACCACCAA | GAGAACCTGGGAGTAGACAAGG |
| *18S* | CGCGGTTCTATTTTGTTGGT | AGTCGGCATCGTTTATGGTC |
