# Supplementary material for: Expression of Adipose MicroRNAs Is Sensitive to Dietary Conjugated Linoleic Acid Treatment in Mice
Source: PLoS One. 2010 Sep 27;5(9):e13005. doi: 10.1371/journal.pone.0013005 (PMC2946340; doi:10.1371/journal.pone.0013005)
Supplement: Text S1 — (0.03 MB DOC) [file pone.0013005.s004.doc]

***Text S4***

***Materials and Methods***

***miRNAs expression analysis***

Total RNA was diluted to 2.5 ng/μl. For each miRNA, 2 μl of these dilutions was reverse-transcribed in 4 μl reaction mix (TaqMan® MicroRNA Reverse Transcription kit, Applied Biosystems) and 1.5 μl of the miRNA-specific reverse-transcription primers provided with the TaqMan® MicroRNA Assay (Applied Biosystems). For the reverse transcription a Perkin-Elmer 9700 Thermal Cycler (PerkinElmer, Wellesley, MA) was used with the following conditions: 16ºC for 30 min; 42ºC for 30 min and 85ºC for 5 min. 2 μl of miRNA-specific cDNA from this reaction was amplified with the TaqMan Universal PCR master mix and the respective specific probe provided in the TaqMan® MicroRNA Assay (Applied Biosystems). The targeted miRNA assay sequences were as follows: *miR-103* 5’-AGCAGCAUUGUACAGGGCUAUGA-3’, *miR-107* 5’- AGCAGCAUUGUACAGGGCUAUCA-3’, *miR-143* 5’-UGAGAUGAAGCACUGUAGCUC-3’, *miR-221* 5’- AGCUACAUUGUCUGCUGGGUUUC-3’*, miR-222* 5’- AGCUACAUCUGGCUACUGGGU-3’. PCR was performed in an Applied Biosystems StepOnePlus^TM^ Real-Time PCR System (Applied Biosystems). Amplification was initiated at 95ºC for 10 min followed by 40 cycles consisting of denaturation at 95ºC for 15 sec and annealing and extension at 60ºC for 1 min. Relative quantification of a target gene was calculated based on efficiency and the crossing point deviation of an unknown sample versus a control, and expressed in comparison to a reference gene used to normalize cDNA (U6 small nuclear RNA). miRNAs expression levels were calculated relative to the values in the respective control group.

***Target mRNA gene expression in adipose tissue***

Real-time polymerase chain reaction (real-time PCR) was used to measure mRNA expression levels of target genes. Aliquots of 0.5 μg of total RNA (in a final volume of 10 μL) were denatured at 90°C for 1 min and then reverse-transcribed to cDNA using MuLV reverse transcriptase (Applied Biosystem, Madrid, Spain) at 42°C for 60 min, with a final step of 5 min at 99°C in a Perkin-Elmer 9700 Thermal Cycler (PerkinElmer, Wellesley, MA). Real-time PCR was performed using the Applied Biosystems StepOnePlus™ Real-Time PCR Systems (Applied Biosystems) with the following profile: 10 min at 95ºC, followed by a total of 40 temperature cycles (15 s at 95ºC and 1 min at 60ºC). Each PCR was performed in a total volume of 6.25 μl, made from diluted cDNA template, forward and reverse primers (1 µM each), and Power SYBER Green PCR Master Mix (Applied Biosystems, CA, USA. Primer sequences are listed in Table S3. All primers were purchased from Sigma (Madrid, Spain). In order to verify the purity of the products, a melting curve was produced after each run according to the manufacturer's instructions. PCR products were also analyzed by electrophoresis in an ethidium bromide-stained agarose gel to check that a single amplicon of the expected size was indeed obtained. The relative quantification of each target gene was calculated based on efficiency and the crossing point deviation of an unknown sample versus a control, and normalized by the expression of the reference housekeeping gene *18S* rRNA [1].

References

1. Pfaffl MW (2001) A new mathematical model for relative quantification in real-time RTPCR. Nucleic Acids Res 29: e45.
